# Supplementary material for: Norcantharidin Suppresses YD-15 Cell Invasion Through Inhibition of FAK/Paxillin and F-Actin Reorganization
Source: Molecules. 2019 May 19;24(10):1928. doi: 10.3390/molecules24101928 (PMC6572169; doi:10.3390/molecules24101928)
Supplement: Supplementary file 1 [file molecules-24-01928-s001.pdf]

molecules

MDPI

Supplementary Materials

Norcantharidin Suppresses YD-15 Cell Invasion Through Inhibition of FAK/Paxillin and F-Actin Reorganization

Kyoung-Ok Hong, Chi-Hyun Ahn, In-Hyoung Yang, Jung-Min Han, Ji-Ae Shin, Sung-Dae Cho * and Seong Doo Hong *

Department of Oral Pathology, School of Dentistry and Dental Research Institute, Seoul National University, Seoul 03080, Korea; hongko95@snu.sc.kr (K.-O.H.); chihyun610@snu.ac.kr (C.-H.A.); inhyoung3@naver.com (I.-H.Y.); 2017-20655@snu.sc.kr (J.-M.H.); sky21sm@snu.ac.kr (J.-A.S.)

* Correspondence: efiwdsc@snu.ac.kr (S.-D.C.); hongsd@snu.ac.kr (S.D.H); Tel.: +82-2-740-8647 (S.-D.C.); Tel.: +82-2-740-8682 (S.D.H.)

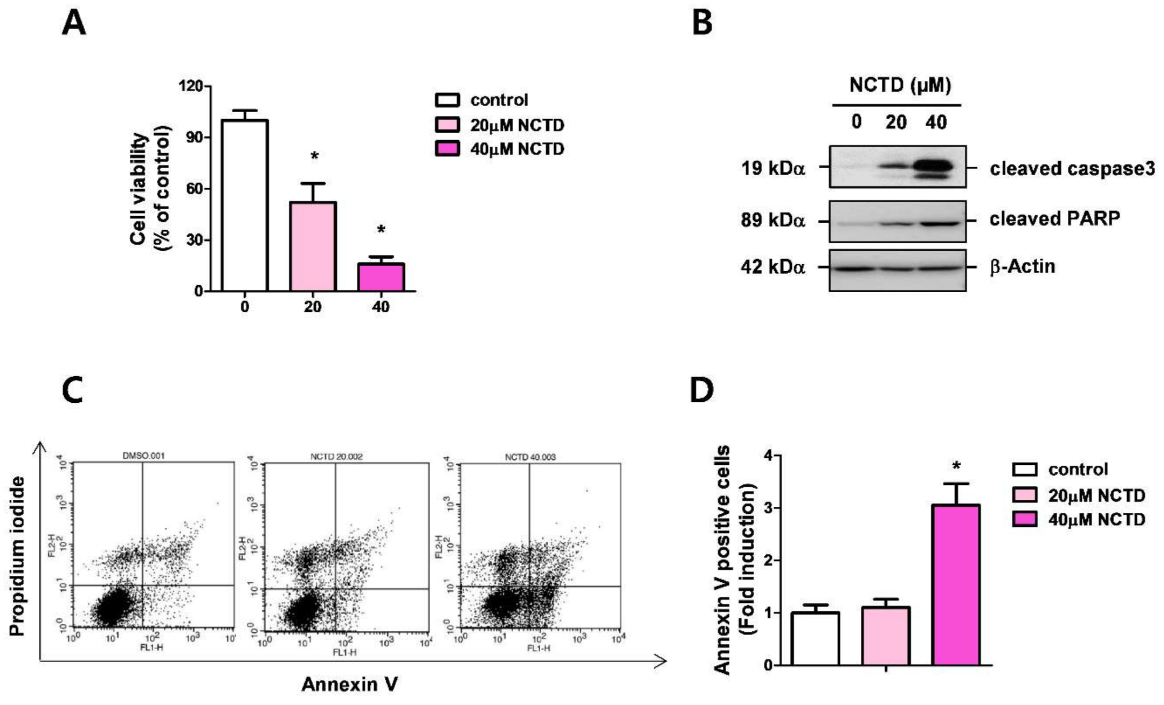

Figure S1. Effects of NCTD on apoptosis in YD-15 cells. (A) Trypan blue exclusion assay for the viability of YD-15 cells after treatment with NCTD (20 and 40 μM) for 24 h. (B) Western blot analysis of the cleavages of caspase 3 and PARP. (C-D) Annexin V/PI double-staining for the detection of apoptosis. Graphs show the mean ± SD of triplicate experiments and significance compared with the vehicle control (*, $p<0.05$).
